# Mismatch repair-deficient hormone receptor-positive breast cancers: Biology and pathological characterization

**DOI:** 10.1186/s12935-021-01976-y

**Published:** 2021-05-17

**Authors:** Elham Sajjadi, Konstantinos Venetis, Roberto Piciotti, Marco Invernizzi, Elena Guerini-Rocco, Svasti Haricharan, Nicola Fusco

**Affiliations:** 1 Division of Pathology, IEO, European Institute of Oncology IRCCS, University of Milan, Via Giuseppe Ripamonti 435, 20141 Milan, Italy; 2 Department of Oncology and Hemato-Oncology, University of Milan, Via Festa del Perdono 7, 20122 Milan, Italy; 3 Physical and Rehabilitative Medicine, Department of Health Sciences, University of Eastern Piedmont, Viale Piazza D’Armi, 1, 28100 Novara, Italy; 4 Department of Tumor Microenvironment and Cancer Immunology, Sanford Burnham Prebys Medical Discovery Institute, 10901 N Torrey Pines Rd, 92037 La Jolla, CA USA

**Keywords:** Breast cancer, Mismatch repair, DNA repair, Immune checkpoint, Immunotherapy

## Abstract

The clinical outcome of patients with a diagnosis of hormone receptor (HR)+ breast cancer has improved remarkably since the arrival of endocrine therapy. Yet, resistance to standard treatments is a major clinical challenge for breast cancer specialists and a life-threatening condition for the patients. In breast cancer, mismatch repair (MMR) status assessment has been demonstrated to be clinically relevant not only in terms of screening for inherited conditions such as Lynch syndrome, but also for prognostication, selection for immunotherapy, and early identification of therapy resistance. Peculiar traits characterize the MMR biology in HR+ breast cancers compared to other cancer types. In these tumors, MMR genetic alterations are relatively rare, occurring in ~3 % of cases. On the other hand, modifications at the protein level can be observed also in the absence of gene alterations and vice versa. In HR+ breast cancers, the prognostic role of MMR deficiency has been confirmed by several studies, but its predictive value remains a matter of controversy. The characterization of MMR status in these patients is troubled by the lack of tumor-specific guidelines and/or companion diagnostic tests. For this reason, precise identification of MMR-deficient breast cancers can be problematic. A deeper understanding of the MMR biology and clinical actionability in HR+ breast cancer may light the path to effective tumor-specific diagnostic tools. For a precise MMR status profiling, the specific strengths and limitations of the available technologies should be taken into consideration. This article aims at providing a comprehensive overview of the current state of knowledge of MMR alterations in HR+ breast cancer. The available armamentarium for MMR testing in these tumors is also examined along with possible strategies for a tailored pathological characterization.

## Background

Every nine seconds, worldwide, a woman is diagnosed with breast cancer, which is by far the most prevalent female tumor and a leading cause of cancer-related death [1]. Approximately two-thirds of these patients have a hormone receptor (HR)+ disease, meaning that the tumor expresses estrogen and/or progesterone receptors [2]. Endocrine therapy (ET), alone or in combination with chemotherapy, and/or targeted therapies, represents the medical treatment backbone in this setting [3]. A significant percentage of patients with HR+ breast cancer, however, eventually develop therapy resistance due to several mechanisms, including tumor immune microenvironment modulation, and mismatch repair (MMR) downregulation [4–7].

The MMR system is an innate form of defense against DNA base mispairing that is essential to human physiology [8]. This highly sophisticated cellular mechanism is modulated by both environmental stimuli and internal processes, resulting in the preservation of the DNA *status quo* [9]. Detrimental modifications in the MMR complex cause genome instability, which is a precondition for cancer to arise [8, 9]. During the past few years, the clinical actionability of MMR alterations has become increasingly important in breast cancer, not only in terms of screening for inherited conditions, but also for patients’ prognostication, prediction of immune checkpoint blockers (ICB) efficacy, and early identification of resistance to therapies [10, 11].

Intrinsic differences seem to characterize the frequency, types, and patterns of MMR alterations in HR+ breast cancers compared to HR− breast cancers and other cancer types [12]. Regrettably, our understanding of the biology that governs the MMR machinery and its clinical actionability in HR+ breast cancer remains incomplete. The substantial lack of tumor-specific guidelines and/or companion diagnostic tests (CDx), further troubles the pathological identification of MMR-deficient (dMMR) cases. In this review, we provide an overview of the specific role of MMR in HR+ breast cancer and discuss the currently available testing strategies for the precise identification of these patients.

## The mismatch repair system machinery

**Fig. 1:**
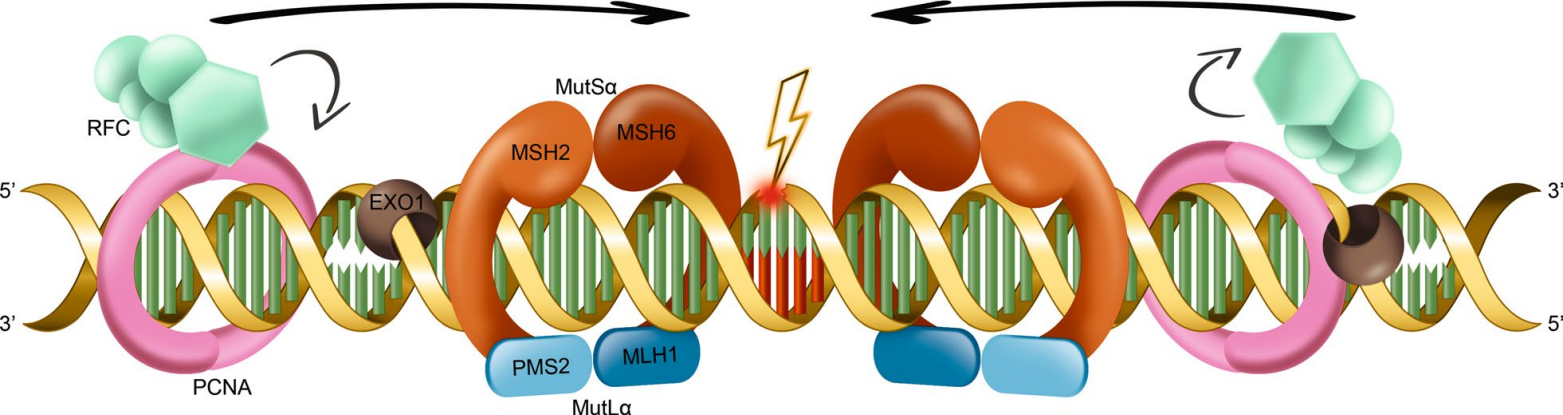
Schematic representation of the MMR system main components. MutSα complex (heterodimer MSH2-MSH6) initiates repair signaling by recognizing the mismatch (thunder). Then, MutLα (heterodimer MLH1-PMS2) is recruited, generating a ternary complex that mediates the downstream processes. Proliferating cell nuclear antigen (PCNA) and replication factor C (RFC) are subsequently activated by MutS. In particular, RFC loads PCNA which is directly implicated in the excision repair and DNA synthesis process. The assembly will initiate endonuclease activity of PMS2 which creates single-strand breaks close to the mismatch and allows for the removal of the wrong-inserted base by exonuclease 1 (EXO1)

Genetic mutations are natural events during DNA replication [13]. Despite not all DNA base mismatches are detrimental, their thorough correction prevents pathogenic mutations from being passed through the cell line [9]. In this respect, the MMR system plays a key role in maintaining genomic integrity and cell homeostasis [14]. The foremost MMR components are mutL homolog 1 (MLH1), mutS homolog 2 (MSH2), mutS homolog 6 (MSH6), mutS homolog 3 (MSH3), post-meiotic segregation increased 2 (PMS2), proliferating cellular nuclear antigen (PCNA) and EXO1 3′→5′ exonucleases [8, 9, 15]. In particular, MSH2 together with MSH6 or MSH3 compose the MutSα and MutSβ heterodimers, respectively, while MLH1 heterodimerizes together with PMS2 to form MutLα [16]. These complexes interact with each other to regulate the recognition and cleavage of incorrect base insertions (Fig. 1) [17]. Malfunction of the MMR system may be responsible for DNA instability, thus promoting tumorigenesis, tumor progression, and resistance to therapies [18, 19]. A collateral phenomenon of MMR-induced genomic instability is represented by microsatellite instability (MSI) [20, 21]. Microsatellites are short tandem repeating sequences of 1–6 nucleotides widely distributed over the DNA, mainly located near the coding region and the ends of chromosomes [22, 23]. Pioneer studies provided evidence suggesting that MSI could be considered, for clinical purposes, as a proxy of the overall genome instability generated by MMR deficiency [24]. Given the elevated frequency of MSI-high (MSI-H) status in dMMR tumors, MMR protein expression and MSI have been historically considered reliable, cost-effective, and (to some extent) interchangeable biomarkers in oncology [25].

## Mismatch repair alterations in HR+ breast cancer

### Frequency and specific pathways involved

**Fig. 2:**
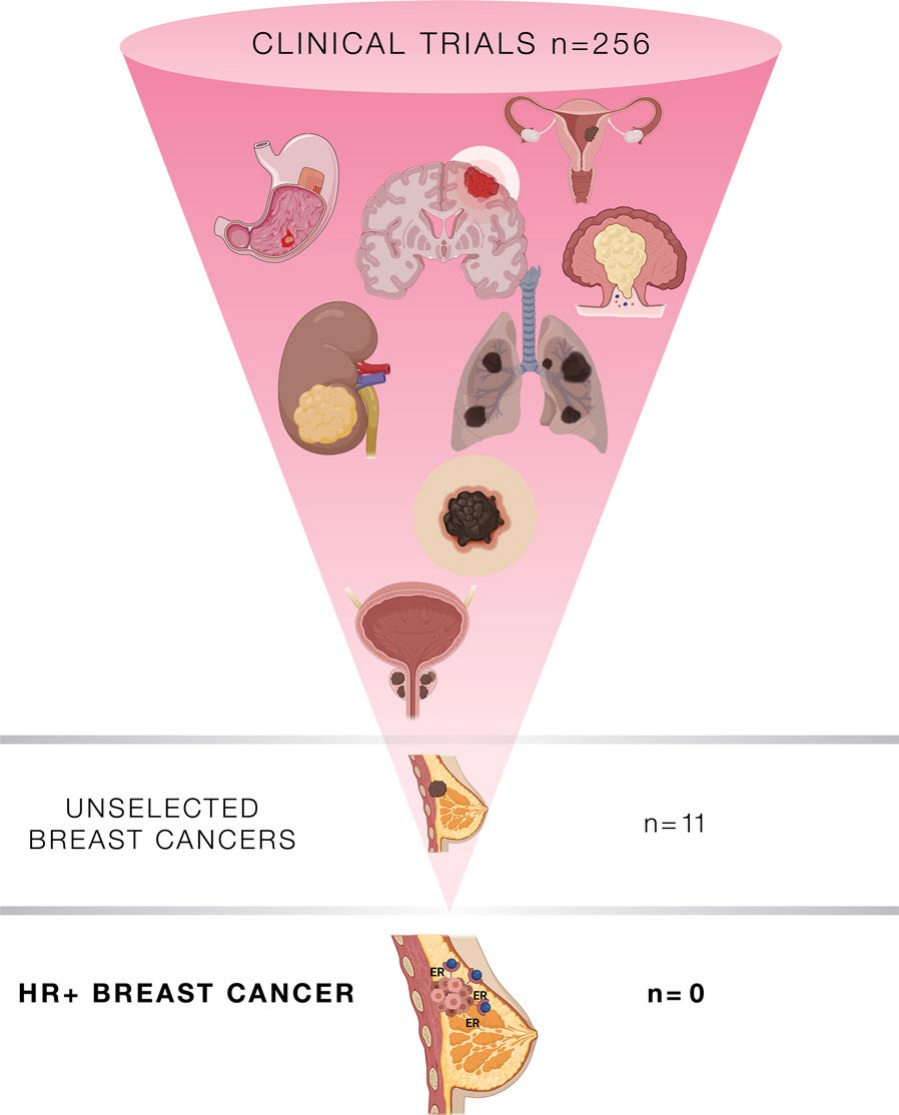
Number of clinical trials based on DNA repair alterations, including MMR deficiency. A total of 256 studies have been conducted on several different tumor types. Among these, only 11 studies involved patients with a diagnosis of breast cancer. None of them included HR+ breast cancers. HR+, hormone receptor-positive

Breast cancers may harbor a wide spectrum of scars in the MMR system, including gene mutations, promoter hypermethylation, and downregulation of RNA levels, as well as alterations to cellular localization of the protein complexes [21, 26–30]. Gene signatures of MMR perturbation have been described in approximately 3 % of these patients, while impaired expression of the MMR proteins seems to be more frequent [31]. Several studies confirmed that MMR deficiency is significantly associated with an increased risk of death in breast cancers, particularly in the HR+ group [21, 29, 32]. Despite this recognized prognostic role, the predictive value of MMR alterations is still controversial in these tumors [33]. Hence, only a few breast cancers were included in the basket trials that led to the histology-agnostic approval of ICB in the presence of MMR deficiency [34]. Of note, none of these patients had a diagnosis of HR+ breast cancer (Fig. 2). Another debated aspect of dMMR HR+ breast cancers is linked to the assumption that the sensitivity to ICB is mainly related to the adaptative immune response against neo-antigens generated by super-mutator cancers [35]. Albeit fitting to some tumor types (e.g. endometrial and colorectal cancers), this model shows certain limitations in HR+ breast neoplasms [36]. Indeed, the tumor mutation burden (TMB) is typically lower in dMMR HR+ breast cancers compared to HR−/HER2− and HER2+ tumors [37, 38].

**Fig. 3:**
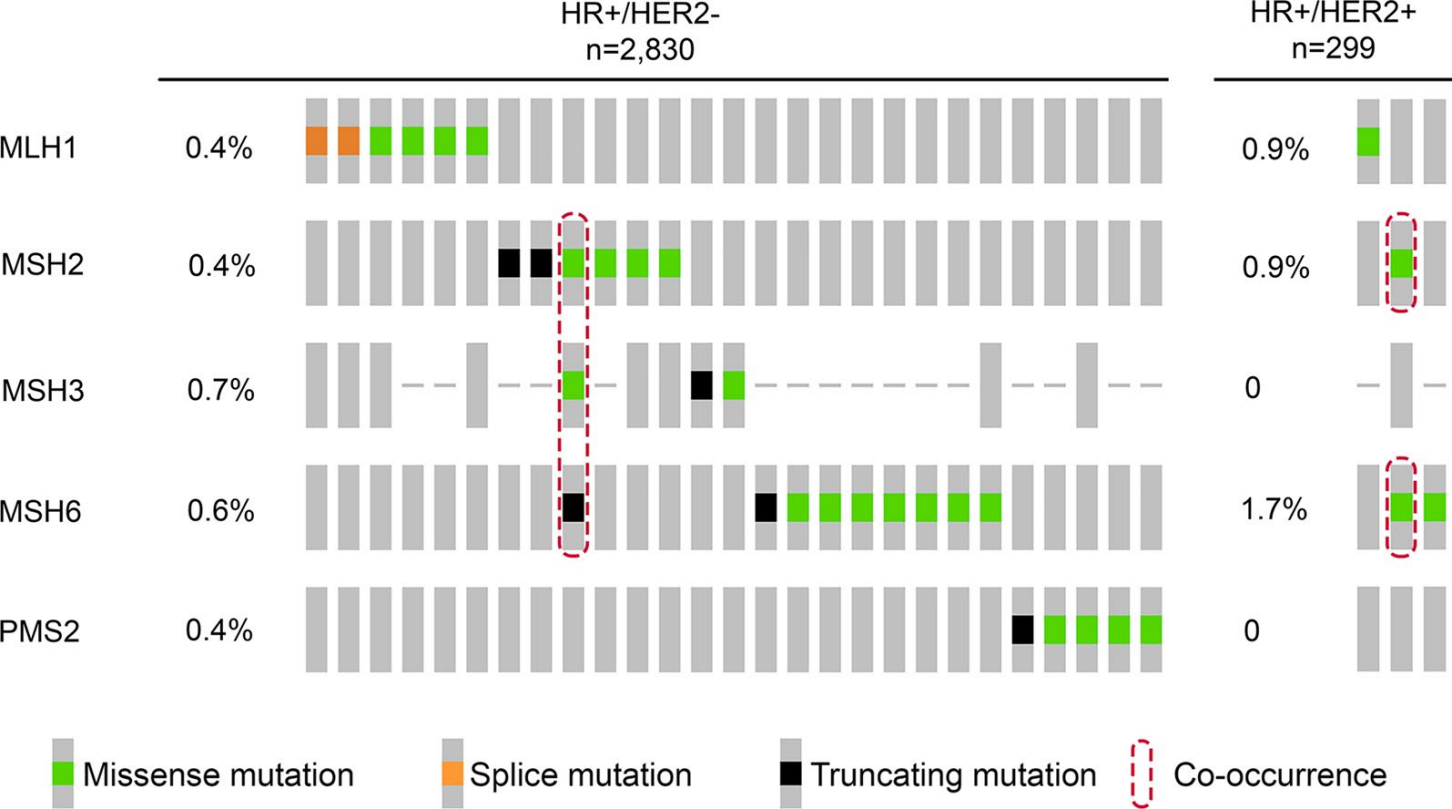
Oncoprint visualization of somatic mutations in the MMR genes across HR+/HER2+ and HR+/HER2- breast cancers.Types of alterations are color-coded on the basis of the legends on the bottom. Each column represents a sample, each row an MMR gene. Tumors included in this analysis have been retrieved from 14 different studies available at cbioportal.org

In HR+ breast cancer, there is a gap of frequency between MMR gene and protein deficiency, where the latter phenomenon is more common than the former [12]. Finding a sound explanation for this remains a subject of controversy, with some authors pointing out suboptimal standard operating procedures in MMR testing and others positing intrinsic biological traits of dMMR HR+ breast cancers [39]. In support of this hypothesis, there is the observation of distinct dMMR phenotypes in HR+ breast cancers. From the proteomic dimension, these tumors are more likely to show a single-marker impairment, preferentially in the MutLα component, unlike in other tumor types (e.g. endometrial and colorectal cancer) that usually show proteins-pair loss [7, 12, 40]. Taken together, the loss of expression at a single-protein level is more commonly observed in HR+ breast cancer compared to HR− (Table 1). More in detail, loss of MLH1 and PMS2 is reported in 7 and 3 % of cases, respectively [30]. The analysis of publicly available next-generation sequencing (NGS) data confirms this propensity, albeit with the aforementioned lower frequency of events, as shown in Fig. 3. Another peculiar trait of breast cancer is represented by the heterogeneous distribution of the MMR+ neoplastic cells. Hence, in more than 13 % HR+ breast cancers (both HER2+ and HER2-), the MMR proteins are heterogeneously expressed inside the tumor without a preferential distribution pattern [21, 41]. The therapeutic implications of this intra-tumor heterogeneity might be critical in regulating intrinsic ET resistance. While the sensitivity of MutL-deficient HR+ breast cancers to ET is decreased, MutS-deficient cases show the contrary [7]. This sort of gene selectivity in dMMR breast cancers might be controlled by cellular signaling pathways that have not been clarified yet. However, the association of MLH1 with ATM activation and the formation of MSH2 complex with ATR/Chk1 activation are events that potentially play a role in these variable ET sensitivities [7, 42]. Of note, a significant portion of HR+ breast cancers shows dysregulation of the phosphoinositide 3 kinase (PI3K)/AKT/mammalian target of rapamycin (mTOR) signaling, which is also the main contributor to ET resistance [43, 44]. Furthermore, actionable co-alterations in PIK3CA and DNA repair genes might lead to the activation of the human leukocyte antigen (HLA) molecules due to increased TMB and subsequent neoantigens production, particularly in the metastatic setting [13, 45, 46]. This subset of patients represents a possible target for immunotherapy with ICB [47].

**Table 1:** Causes of MMR perturbation at the protein expression level in breast cancer according to the hormone receptor status

	HR+ (n = 69)	HR− (n = 12)
MutS alone, n (%)		
MSH2	17 (25)	3 (25)
MSH6	4 (6)	0
Both proteins	6 (9)	0
MutL alone, n (%)		
MLH1	10 (15)	1 (8)
PMS2	3 (4)	0
Both proteins	2 (3)	1 (8)
MutS + MutL, n (%)		
MutS + MLH1	4 (6)	0
MutL + MSH2	2 (3)	1 (8)
MutL + MSH6	1 (2)	0
MSH2 + MLH1	8 (12)	4 (3)
MSH2 + PMS2	1 (2)	1 (8)
MSH6 + MLH1	2 (3)	0
MSH6 + PMS2	1 (2)	0
All proteins	8 (12)	1 (8)

### Clinical implications

Defects in specific components of the MMR system can causally induce ET resistance in HR+ breast cancer and thereby, poor patient outcomes [7]. However, MMR deficiency also appears to uncouple hormone signaling and cell cycle regulation at the G1/S cell cycle transition, thereby potentially making these tumors more susceptible to cyclin-dependent kinase (CDK)4/6 inhibition, even as a front-line therapy [7, 48]. Interestingly, it has been previously reported that a possible mechanism of chemoresistance in HR+/HER2-transformed breast cancer is related to MSH2 downregulation by TGFβ-induced miR-21 [49]. In addition, preclinical data suggest that the DNA homologous recombination gene BRCA1 may be a positive regulator of MSH2 in HR+/HER2+ breast cancer, suggesting that its tumor-suppressor function can be mediated by the MMR system [50]. In tamoxifen-treated HR+ breast cancer patients, MMR deficiency is related to worse overall and disease-specific survival (HR 2.29, 95 % CI 1.02–5.17, *p* = 0.040 and HR 2.71, 95 % CI 1.00–7.35, *p* = 0.042, respectively). This finding suggests a potential role of MMR status to detect HR+ patients who may benefit from treatments other than ET [29]. In another study, the overall survival (OS) rate of dMMR and non-dMMR tumors were profoundly different in both Luminal B and HR- breast cancers. Accordingly, patients with HR+ dMMR carcinomas had worse OS (median = 77 months, range = 0–115 months) than those with MMR-proficient (pMMR) or MMR-heterogeneous (hMMR) tumors (median = 84 months, range = 0–127 months) (*p* = 0.008). In contrast, HR− dMMR patients treated with chemotherapy showed a better OS compared to the pMMR or hMMR group (median = 87 and 79 months, range = 73–123 and 8–113 months, respectively; *p* < 0.001) [21]. Likewise, the potential effect of each MMR protein has also been studied in different subtypes of breast cancer. In this regard, the most recurrent type of MMR deficiency both in HR+ and HR− breast cancers is related to the loss of expression of MSH2 alone, with a significant correlation with shorter survival times (*p* = 0.04) [30]. As discussed above, although dMMR colorectal and endometrial tumors demonstrate a response to ICB, whether dMMR breast tumors would do so as well remains untested. This is an interesting question for further study because of the apparent innate differences in the impact of MMR deficiency on TMB in HR+ breast cancers relative to that observed in colorectal or endometrial cancers [37, 51]. A final point of interest to improve actionability against this driver of poor outcomes in HR+ breast cancer is to better understand the biological and functional impact of mutations in MMR genes that are observable in the neoplastic cells. The vast majority of these are individual missense mutations that, at this time, remain variants of unknown significance.

### Genetic risk

The association of germline variants in MLH1, PMS2, MSH2, and MSH6, also referred to as Lynch syndrome genes, with breast cancer risk remains controversial. Unlike in the canonical Lynch-syndrome tumors, MMR deficiency is sporadic in the vast majority of HR+ breast cancer, with only 0.2–0.5 % of cases being classified as syndromic [52]. Consensus in the literature is that there is no statistically significant association between germline mutations in these MMR genes and breast cancer risk [53]. However, some studies do identify an increased risk of breast cancer incidence, and younger age at diagnosis in women with germline variants in PMS2 and MSH6, but no association with germline mutations in MLH1 or MSH2 [54, 55]. A recent study conducted in a cohort of 711 patients with hereditary breast cancer, reported that 69 (9.7 %) patients had at least one germline mutation in the MMR genes. In 32 (4.5 %) of them, these mutations were defined as pathogenic or likely pathogenic [56]. Furthermore, recurrent germline mutations of MLH1 V384D, in the absence of high-TMB, were found in ~ 14 % of HR+/HER2+ breast cancers in East Asian patients, suggesting that in this subset of neoplasms MLH1 haploinsufficiency is more likely to contribute to tumor predisposition factor rather than to constitute a direct oncogenic driver [57].

### Interplay between MMR and the anti-tumor immune response

There are accumulating data on the interaction between MMR and other immune-related biomarkers that can be employed in the next future to improve the tailored clinical management of HR+ breast cancer. Lately, it has been shown that dMMR breast cancers are related to high tumor-infiltrating lymphocytes (TILs) counts (median of 5, interquartile range 1–10) compared to pMMR tumors (median of 1, *p =* 0.009 by Mann–Whitney test) [29]. It has been also indicated that dMMR breast cancers show significantly higher expression of programmed death-ligand 1 (PD-L1) and CD8 (69 and 62 %, respectively, n = 13) than those with intact MMR expression (35 and 29 %, respectively, n = 285) [41]. Another group performed an IHC analysis for CD3, CD4, and CD8 expression on both HR+ and HR− breast cancers [58]. Interestingly, the Authors observed T-cell predominance together with high TILs in 50 % (n = 2/4) dMMR tumors (range 4–175, per 10 high-power fields). Large multicentric clinical and translational studies specifically designed to include HR+ breast cancers are warranted not only for revealing novel biomarkers but also for better understanding the relationship between MMR and the other traditional biomarkers.

## Mismatch repair testing: focus on HR+ breast cancer

### Rationale

**Fig. 4:**
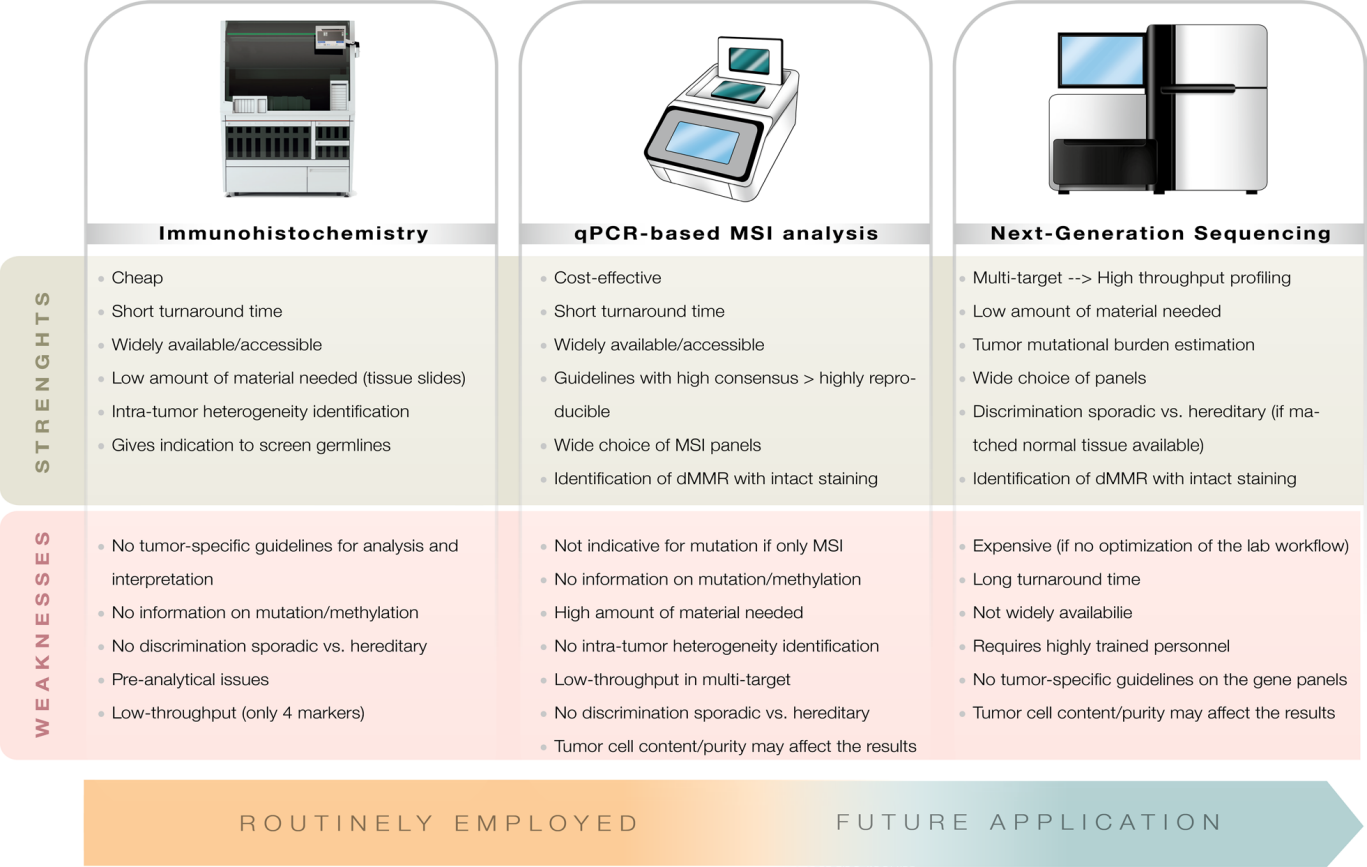
Currently available technologies for MMR status assessment. Diagramshowing the strengths and weaknesses of each method for the analysis in breast cancer samples. *qPCR* quantitative PCR, *MSI* microsatellite instability, *IHC* immunohistochemistry

The pathological identification of dMMR breast cancers has proven to be extremely challenging due to the constraints of the existing methods, and the absence of CDx tests and/or tumor-specific guidelines [12]. So far, the variety of currently available locally developed laboratory tests have been shaped on those approved for colorectal and endometrial carcinomas [59, 60]. For a long time, there has been a nihilistic view of the actual clinical utility of MMR screening for HR+ breast cancer, probably because of the relatively low frequency of the dMMR phenotype in these patients. Lately, the histology-agnostic approval of ICB immunotherapy with pembrolizumab -an anti-programmed cell death-1 (PD-1) drug- for unresectable or metastatic dMMR or MSI-H solid tumors, increased the importance of assessing MMR status in breast cancer [61]. Yet, the likely-predictive and prognostic value of MMR fortifies its role as a promising biomarker to improve clinical decision-making for breast cancer patients [21, 29]. Reference methods for MMR profiling depend on IHC for the four main MMR proteins with or without sequencing assays directed towards selected microsatellite markers (e.g. Bethesda panel and MSI Analysis System) [25, 62]. Despite their reliability, these diagnostic strategies have several limitations, including the relatively low sensitivity in breast cancer due to their heterogeneous protein expression [63–65]. To overcome these issues, new molecular-based methods such as novel real-time PCR (RT-PCR) panels and droplet digital PCR (ddPCR)-based assays, as well as NGS panels have recently emerged (Fig. 4) [19, 66].

### Immunohistochemistry

Mutations in MLH1, MSH2, MSH6, and PMS2 may result in proteolysis of the MMR heterodimers and subsequent nuclear loss of protein immunoexpression [67]. Due to its reliability, cost-effectiveness, and the large availability, IHC is usually considered a pillar as a first-line MMR testing method [10]. Hence, pre-diluted monoclonal antibodies against MLH1, MSH2, MSH6, and PMS2 are commonly accessible in the vast majority of pathology laboratories [24]. Given the intra-tumor heterogeneity of MMR protein expression in HR+ breast cancer, the reliability and reproducibility of complementary and/or surrogate biomarkers have been investigated by several research groups. Recently, the assessment of phosphatase and tensin homolog (PTEN) expression, a key tumor suppressor gene mainly involved in DNA repair, apoptosis, and cell survival, was suggested as a complementary biomarker to pre-screen MMR status in breast cancer [30, 68]. According to this workflow, the positive predictive value of PTEN retained status for pMMR ranged from 94.6 % in HR+ tumors to 100 % in HER2-enriched and HR − breast cancers. Moreover, a significant association of the MMR status with PTEN IHC was seen in the HR+ cluster (*p <* 0.0001, Fisher’s exact test) suggesting that alterations in PTEN expression are significantly related to MMR deficiency [69]. However, it has been recently demonstrated that, in breast cancer, MMR protein loss is more frequently detected compared to MSI, suggesting a lack of interchangeability of these two tests [21]. Importantly, not all MMR proteins evenly influence either mutational load or MSI when deficient [30, 70]. The potential impact of technical artifacts and/or intra-tumor heterogeneity phenomena on MMR status assessment still needs to be fully elucidated [71]. Albeit MMR IHC in breast cancer is being suggested as a valid clinical test by different studies, several issues including the use of specific antibody clones, CDx, and/or interpretation guidelines have still to be addressed. Other major drawbacks of this technique are related to the lack of specific recommendations on cold ischemia time, fixation protocols, primary antibody clones, concentrations, and staining platform as well as interpretation guidelines [72].

### qPCR-based MSI analysis

MSI analysis has been originally performed by RT-PCR for five microsatellite markers, consisting of three dinucleotides (i.e. D2S123, D5S346, and D17S250) and two mononucleotides (i.e. BAT-25 and BAT-26) repeats [73–75]. This panel, however, as recommended by the revised Bethesda Guidelines, has proven sufficiently high sensitivity and specificity only in the detection of syndromic tumors [76, 77]. Novel PCR panels (e.g. MSI Analysis System, Promega®) targeting mononucleotide repeats have been proposed as reliable alternative options to the Bethesda one [78]. Lately, new high-performance assays have been proposed as viable and complementary options to IHC and standard RT-PCR panels, including PlentiPlex™ MSI (Pentabase), OncoMate™ (Promega), Idylla™ MSI Test (Biocartis), TrueMark (Thermofisher), and Bio-Rad ddPCR [79]. However, no data are currently available for their reliability in breast cancer. The major limitation of RT-PCR assays is that insufficient tumor content may not allow the detection of MSI and/or alterations in the sequence target. Furthermore, in tumors with low MSI/dMMR frequency, such as breast cancer, little data are available and the exploitation of IHC and MSI RT-PCR protocols is highly questioned [12]. Of note, epigenetic silencing of MLH1 by promoter hypermethylation is a crucial event that may lead to MMR deficiency. Methylation-specific PCR for the 5′ CpG promoter of MLH1 is primarily carried out for the assessment of this condition [80].

### Next-generation sequencing

Recently, NGS has appeared as an ultra-sensitive method to characterize MSI and MMR status accurately and simultaneously [81]. Thereby, NGS-based methods demonstrated higher performances when compared to previous technologies and are potentially useful to expand MSI testing, particularly in those cancers characterized by lower MSI-H/dMMR frequencies [82]. Indeed, NGS panels can screen a larger number of microsatellite loci compared to RT-PCR [83]. This allows parallel high-throughput analysis of both microsatellites and genes leading to the simultaneous identification of other actionable alterations. Interestingly, MSI testing performed using NGS can be easily integrated with other relevant biomarkers such as TMB, using targeted-specific panels and avoiding the costs of whole-exome or whole-genome sequencing [84, 85]. To date, NGS is rarely performed in breast cancer due to its higher cost compared to lower throughput methods, and the lack of tumor-specific panels of genes which could reveal a potential association of MMR deficiency with other clinically actionable genes [66]. It is important to keep in mind that current NGS genomic diagnoses of MMR deficiency through TMB, genomic scars, or MSI were developed based on canonical Lynch syndrome cancers (colorectal predominantly) [86]. To ensure high specificity and sensitivity, these diagnostic strategies might need to be re-developed in the context of breast cancer. Considering all the pros and cons, although the aforementioned methods represent candidate tools for the establishment of promising MMR testing strategies, they require profound experimentation before being implemented in clinical setting.

## Concluding remarks

Alterations of the MMR system are relatively rare events in HR+ breast cancer. However, due to the extremely high frequency of this neoplastic condition in the female population worldwide, MMR deficiency is of great clinical interest. The prognostic value of MMR deficiency coupled with its predictive role for ICB and ET resistance further increases the significance of performing this analysis in HR+ breast cancers. From the diagnostic perspective, it is crucial to adopt a tailored methodology to cover all of the intrinsic characteristics of breast cancers that are usually not shared by other types of tumors where MMR deficiency is more common. The versatility and efficiency of RT-PCR, combined with its cost-effectiveness, sensitivity, and specificity, facilitate the adoption of this technology in virtually all molecular pathology laboratories. On the other hand, NGS panels allow covering several different alterations simultaneously, even starting from low input DNA. Although the rough costs of this technology, which requires specialized centers with highly trained personnel, are relatively high, the optimization of the laboratory workflow allows for a favorable cost-benefit ratio. On the other hand, pathologists, molecular biologists, and clinicians should be fully aware of the lack of interchangeability between MMR protein expression profiling and gene sequencing in HR+ breast cancers. It is important to remark that in the case of negative results at DNA analysis, a proteomic technique should be performed to avoid false-negative results. Further translational research and clinical studies coupled with tumor-specific guidelines for analytical and preanalytical phases are warranted to precisely characterize MMR status in HR+ breast cancer.

## Data Availability

Not applicable.

## Abbreviations

HR: Hormone receptor
MMR: Mismatch repair
ICB: Immune checkpoint blockers
CDx: Companion diagnostic tests
dMMR: MMR-deficient
MLH1: MutL homolog 1
MSH2: MutS homolog 2
MSH6: MutS homolog 6
MSH3: MutS homolog 3
PMS2: Post-meiotic segregation increased 2
PCNA: Proliferating cellular nuclear antigen
MSI: Microsatellite instability
MSI-H: MSI-high
TMB: Tumor mutation burden
NGS: Next-generation sequencing
PI3K: Phosphoinositide 3 kinase
mTOR: Mammalian target of rapamycin
HLA: Human leukocyte antigen
CDK: Cyclin-dependent kinase
OS: Overall survival
pMMR: MMR-proficient
hMMR: MMR-heterogeneous
TILs: Tumor-infiltrating lymphocytes
PD-1: Programmed cell death-1
PD-L1: Programmed death-ligand 1
IHC: Immunohistochemistry
RT-PCR: Real-time PCR
ddPCR: Droplet digital PCR
PTEN: Phosphatase and tensin homolog
